# Zengye Decoction Attenuated Severe Acute Pancreatitis Complicated with Acute Kidney Injury by Modulating the Gut Microbiome and Serum Amino Acid Metabolome

**DOI:** 10.1155/2022/1588786

**Published:** 2022-05-09

**Authors:** Xiao-Yu Dai, Qian Hu, Jia-Qi Yao, Xiao-Jia Wu, Yi-Fan Miao, Juan Li, Mei-Hua Wan, Wen-Fu Tang

**Affiliations:** Department of Integrated Traditional Chinese and Western Medicine, West China Hospital, Sichuan University, Chengdu 610041, Sichuan Province, China

## Abstract

**Objective:**

To explore the effect and underlying mechanism of Zengye decoction (ZYD), a traditional formula from China, on the severe acute pancreatitis (SAP) rat model with acute kidney injury (AKI).

**Methods:**

The SAP-AKI model was induced by 3.5% sodium taurocholate. Rats were treated with normal saline or ZYD twice and sacrificed at 36 h after modeling. Amylase, lipase, creatinine, blood urea nitrogen, kidney injury molecule 1(KIM-1), and multiple organs' pathological examinations were used to assess the protective effect of ZYD. Gut microbiome detected by 16S rRNA sequencing analysis and serum amino acid metabolome analyzed by liquid chromatography-mass spectrometry explained the underlying mechanism. The Spearman correlation analysis presented the relationship between microflora and metabolites.

**Results:**

ZYD significantly decreased KIM-1(*P* < 0.05) and the pathological score of the pancreas (*P* < 0.05), colon (*P* < 0.05), and kidney (*P* < 0.05). Meanwhile, ZYD shifted the overall gut microbial structure (*β*-diversity, ANOSIM *R* = 0.14, *P*=0.025) and altered the microbial compositions. Notably, ZYD reduced the potentially pathogenic bacteria—Bacteroidetes, Clostridiales vadin BB60 group, and uncultured_Clostridiales_bacterium, but promoted the short-chain fatty acid (SCFA) producers—Erysipelotrichaceae, Bifidobacterium, Lactobacillus, and *Moryella* (all *P* < 0.05). Moreover, principal component analysis (PCA), partial least squares-discriminant analysis (PLS-DA), and hierarchical clustering analysis (HCA) presented a remarkable change in amino acid metabolome after SAP-AKI induction and an apparent regulation by ZYD treatment (R2Y 0.878, *P*=0.01; Q2 0.531, *P*=0.01). Spearman's correlation analysis suggested that gut bacteria likely influenced serum metabolites levels (absolute *r* > 0.4 and FDR *P* < 0.02).

**Conclusions:**

ZYD attenuated SAP-AKI by modulating the gut microbiome and serum amino acid metabolome, which may be a promising adjuvant treatment.

## 1. Introduction 

Severe acute pancreatitis (SAP) is a changeable and possibly lethal disease with multiple organ dysfunctions [1]. Acute kidney injury (AKI) is a frequent complication of SAP with extremely high mortality [2]. Metabolic reprogramming is a part of accepted pathologies underlying SAP-AKI, but the exact mechanism remains unclear [3]. Meanwhile, unique treatment for SAP-AKI is still under exploration [4].

Recently, the gut microbiome-modulating endogenous metabolism has aroused many interests [5]. More and more research studies have indicated that gut bacterial dysbiosis plays a vital role in the pathological mechanism of acute pancreatitis (AP) and AKI [6, 7]. Also, reports have demonstrated that improving gut microbiota could protect against SAP [8, 9]. However, rare studies investigated the effect of modulating microbiome on SAP-AKI. The serum metabolome is responsive to the gut microbiome variation [10], and metabolomics is a powerful tool to explore potential pathogenesis and effective drugs for diseases [11]. Amino acids serve as major nutrients and signaling molecules to regulate various physiological processes [12]. Nevertheless, AP and AKI cause distinct disorders in the amino acid metabolic profile [13, 14]. Several studies presented that regulating the overall serum metabolome may protect AP [15–17], but very little is known about the effect of regulating the amino acid metabolome in SAP-AKI.

Zengye decoction (ZYD), a traditional Chinese medicine, consists of *Scrophulariae* (Xuanshen), *Ophiopogonis* (Maidong), and *Rehmannia* (Shengdi), which has been widely used in many Asian countries for thousands of years [18]. Research studies have reported that ZYD can ameliorate metabolic disorders like diabetes [19, 20]. Remarkably, Liu et al. proved that ZYD could regulate the gut microbiota and amino acid metabolism pathway to cure constipated rats [21]. To our knowledge, no report has explored the application of ZYD on SAP or AKI. Thus, we hypothesized that ZYD could protect SAP-AKI by modulating the gut microbiome and serum amino acid metabolome. This study may provide a novel therapeutic method for SAP-AKI and elucidate the potential underlying mechanisms.

## 2. Materials and Methods

### 2.1. Animals

Twenty-one male Sprague Dawley rats (weight: 220 ± 10 g, clean grade) were obtained from Dashuo Experimental Animal Co., Ltd (Chengdu, China) (certificate no. 512003500015140; license no. SCXK (Sichuan) 2020–030). After one week of acclimation, the animals fasted but were free to access water 24 h ahead of the experiment. The experimental protocol passed the ethics of the West China Hospital of Sichuan University and was approved by the Animal Ethics Committee (protocol number: 2020234A, Chengdu, China).

### 2.2. ZYD Preparation

ZYD decoction is composed of Scrophulariae, Ophiopogonis, and Rehmannia. According to the Chinese Pharmacopoeia, the appropriate daily dose of these crude drugs for an adult (60 kg) is 15 g, 12 g, and 12 g, respectively. Besides, the frequently used administration of this decoction is 3 times a day. Thus, a single dose per kilogram of body weight is about 0.21 g/kg (=0.021 g/100 g). In line with the experimental methodology of pharmacology written by Xu et al. [22], a 6.3-fold dose of an adult is reasonable for Sprague Dawley rats, which is about 0.13 g/100 g body weight.

These crude drugs were obtained from the Affiliated Hospital of Chengdu University of Traditional Chinese Medicine (Chengdu, China), where they were processed to spray-dried particles by professionals in the pharmacy department after identification. Afterward, we reconstituted the spray powder with 40°C distilled water at 0.13 g/ml and treated the ZYD group experimental animals by intragastric administration (1 ml/100 g body weight).

### 2.3. Experimental Design

Sprague Dawley rats were randomly separated into the control group (C, *N* = 7) with sham operation, SAP model group (MG, *N* = 7), and ZYD treatment group (ZYD, *N* = 7). All rats were anesthetized with pentobarbital sodium solution (2%) by intraperitoneal injection (50 mg/kg) [23]. The subsequent operation is similar as Zhang et al. described in their research [24]. In brief, the biliopancreatic duct was found and carefully cannulated, and then, a microvascular clamp was applied to temporarily close the hepatic duct. Next, 3.5% sodium taurocholate (1 ml/kg body weight) induced the SAP model by infusion at a speed of 6 ml/h. Finally, we replaced the pancreas and cautiously closed the abdomen. ZYD decoction was applied to experimental rats at 12 h and 24 h after SAP induction by intragastric injection, respectively. At the same time, the C and MG were administered equivalent volumes of saline. Rats were sacrificed at 36 h after the SAP model establishment. Blood samples stood for 2 h before centrifugation (1,300 g, 10 min, 4°C), and serum samples were stored at −80°C until analysis. Fresh tissues, including pancreas, colon, and kidney, were fixed with paraformaldehyde at room temperature and sent to Lilai Biotechnology Company for embedding by paraffin and section. Fresh fecal samples taken from the colon were rapidly preserved in a liquid nitrogen container and maintained at −80°C until analysis.

### 2.4. Laboratory Tests

The concentrations of amylase, lipase, creatine (Cr), and blood urea nitrogen (BUN) in serum were detected by Roche Cedex C501 automatic biochemical analyzer (Switzerland). Serum kidney injury molecule 1 (KIM-1) level was measured by ELISA Kit (Cat. No. ZC-37184) from Zhuo Cai Technology Company (Shanghai, China) in line with the instructions from the manufacturer.

### 2.5. Histopathologic Examination

The paraffin-embedded pancreas, colon, and kidney tissues from each group, after sliced (5 *μ*m), dewaxed, and stained with hematoxylin and eosin (H&E), were observed under an upright microscope (Zeiss, Germany) by two professional pathologists in a blind manner. The pancreas (×200) and kidney (×200) were scored, respectively, for edema, neutrophil infiltration, necrosis, and hemorrhage on a 0 (none) to 4 (severe) scale [25]; then, the composite scores were calculated. The colon (×200) was scored for inflammation-associated histological changes using an established scoring system with a scale from 0 to 4 [26]. Random ten fields of each section were counted, and the average of the composite scores for each field was presented as the final pathological injury score.

### 2.6. 16S rRNA Sequencing Analysis of Gut Microbiome

#### 2.6.1. DNA Extraction

The stools were sent to OE Biotech (Shanghai, China) to perform the 16S rRNA analysis. According to the instructions from the manufacturer, overall genomic DNA was extracted through DNeasy PowerSoil Kit (QIAGEN, cat. no. 12888, USA). NanoDrop (Thermo Fisher 2000, USA) and agarose gel examined the concentration of DNA. Then, they were applied for PCR amplification with the aid of barcoded primers and Tks Gflex DNA Polymerase (Takara, cat. no. R060B, Japan).

Amplifying the particular regions (V3–V4) of 16S rRNA genes helped the bacterial diversity analysis and the widespread primers: 343F (5′-TACGGRAGGCAGCAG-3′) and 798R (5′- AGGGTATCTAATCCT-3′) were used in this study. After surveying the quality utilizing gel, purified by AMPure XP beads (Agencourt, USA), the PCR products were amplified for PCR again. Then, the final amplicon was acquired by purifying again and quantified utilizing the Qubit dsDNA assay kit (Thermo Fisher, cat. no. Q32854, USA). In the end, these amplicons were merged at equal amounts for subsequent sequencing.

#### 2.6.2. Bioinformatic Analysis

Unprocessed sequencing data were saved in the FASTQ format. Trimmomatic software (version 0.35) was applied to preprocess the paired-end reads for detecting and cutting off blurred bases (N) [27]. The sliding window trimming method helped cut out the low-quality sequences (average quality score <20). Then, Flash software (version 1.2.11) assembled paired-end reads [28]. Parameters in the assembly were as follows: 10 bp–200 bp of overlapping and 20% of maximum mismatch rate. QIIME software (version 1.8.0) assisted in further denoising of sequences as below: abandoning reads with sequences that were blurred, homologous, or below 200 bp; retaining reads whose 75% bases are above Q20. Then, reads with chimera were explored and deleted [29]. With the help of VSEARCH software (version 2.4.2), operational taxonomic units (OTUs) were generated from the clean reads, which were derived from primer sequence removal and clustering (similarity cutoff: 97%) [30]. The representative read of each OTU was picked by the QIIME package. Analysis for *α*-diversity, such as Shannon index, Simpson index, Chao 1 index, and observed species, was detailed in previous research [31]. Linear discriminant analysis (LDA) of effect size (LEfSe) was performed according to Zhu et al. [32]. Nonmetric multidimensional scaling (NMDS) based on Bray-Curtis distance, analysis of similarities (ANOSIM), and Kyoto Encyclopedia of Genes and Genomes (KEGG) pathway analysis was described in the research implemented by Lei et al. [33, 34]. Ribosomal Database Project (RDP) classifier was utilized to annotate all typical reads against the SILVA database (version 123) with a 70% confidence threshold [35].

### 2.7. Serum Amino Acid Metabolome Detection

For metabolomic analysis, twenty-one serum samples were sent to the West China-Washington Mitochondria and Metabolism Research Center. A merged method for targeted analysis of amino acids and untargeted profiling was implemented in this study [36]. The UltiMate 3000 rapid separation liquid chromatography (Thermo Fisher Scientific, USA) equipped with a BEH Amide column (100 × 2.1 mm, 1.7 *μ*m, Waters, USA) coupled with *Q* Exactive Plus quadrupole-Orbitrap high-resolution mass spectrometry (Thermo Fisher Scientific, USA) performed this measurement. Detailed procedures from reagent preparation to liquid chromatography-tandem mass spectrometry (LC-MS/MS) data analysis were depicted in the research reported by Zhang et al. [37]. Special parameters in this study were as follows. The column temperature was 35°C, and the elution gradient linearly changed: 0–2 min, 100% B; 2–4 min, 100%–95% B; 4–9 min, 95%–85% B; 9–14 min, 85%–50% B; 14–17 min, 50%–50% B; 17–17.1 min, 50%–100% B; and 17.1–25 min, 100% B. Differentially expressed metabolites were screened under these conditions: 1. Kruskal-Wallis test *P* < 0.05; 2. variable importance for the projection (VIP) score >1. R software (version 4.1.0) was used for statistical data analysis, such as Kruskal-Wallis test, hierarchical clustering analysis (HCA), principal component analysis (PCA), partial least squares-discriminant analysis (PLS-DA), and pathway analysis against the database KEGG [38].

### 2.8. Spearman's Correlation Analysis

The cor.test (R software 4.1.0) performed Spearman's correlation analysis between serum metabolite concentration and genera abundance. The *P*-value of multiple comparisons was corrected by Benjamin-Hochberg false discovery rate (FDR), and the association was considered statistically significant if absolute *r* value > 0.4 and adjusted *P* < 0.2 [39].

### 2.9. Statistical Analysis

Data (mean ± standard error of mean (SEM)) were analyzed by SPSS26.0 (Chicago, IL, USA). The type of parametric distribution was examined using the Shapiro-Wilk test. One-way ANOVA with post hoc least significant difference (LSD) test was carried out for three groups with standard distribution data. Mann-Whitney *U* test for two groups and Kruskal-Wallis test for three groups were used to compare continuous variables. *P* < 0.05 was regarded as statistically significant.

## 3. Results

### 3.1. ZYD Showed a Protective Effect against SAP-AKI

**Figure 1:**
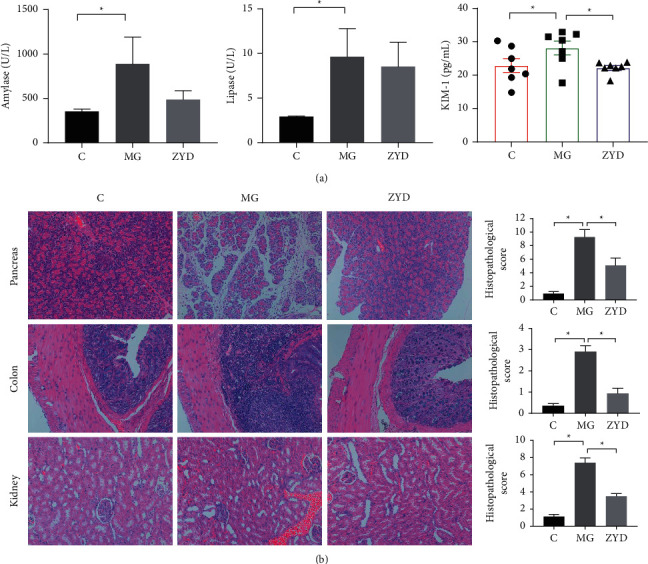
ZYD protects against SAP-AKI. (a) Comparison of amylase, lipase, and kidney injury molecule 1(KIM-1) in serum. ^*∗*^: *P* < 0.05 (amylase and lipase decided by the Mann-Whitney test, KIM-1 decided by the one-way ANOVA followed LSD test). (b) Pathological picture and scores of the pancreas, colon, and kidney. Scale bar: 100 *μ*m (×200). ^*∗*^: *P* < 0.05 (one-way ANOVA followed LSD test). (c) Healthy control group with the sham operation, MG: severe acute pancreatitis model group, and ZYD: Zengye decoction treatment group. *n* = 7 (per group). Data are presented as the mean ± SEM.

In this experiment, we established the SAP-AKI model by a refusion of 3.5% sodium taurocholate and sacrificed rats at 36 h after modeling to observe the effect of ZYD. Serum amylase (*P* < 0.05), lipase (*P* < 0.05), and KIM-1 (*P* < 0.05) levels significantly increased after SAP induction. Conversely, ZYD decreased the serum concentration of amylase and lipase and significantly reduced KIM-1 (*P* < 0.05) (Figure 1(a)). There was no distinct difference in Cr and BUN among the three groups (data not shown).

The MG group showed severe morphological injuries like edema and acinar cell necrosis in the pancreas, neutrophil infiltration in the colon, and hemorrhage in the kidney compared to the C group (*P* < 0.05, Figure 1(b)). In contrast, a significant injury amelioration of the pancreas (*P* < 0.05), colon (*P* < 0.05), and kidney (*P* < 0.05) was presented in the ZYD group (Figure 1(b)). In brief, these results suggest a protective effect from ZYD on SAP-AKI.

### 3.2. ZYD Modulated the Gut Microbiome in SAP-AKI

To detect the effect of ZYD on gut microflora in rats with SAP-AKI, we analyzed 21 fecal samples using the 16S rRNA gene sequencing method. OTUs, clustered from the high-quality amplicon sequence variants from the gut bacterial gene V3-V4 region, were the basis for gut microbiome comparison. A total of 3,083 OTUs overlapped among the three groups, with 143, 157, and 117 OTUs specifically detected in the C, MG, and ZYD groups, respectively (Supplementary Figure 1(a)). The species accumulation curves tended to flatten out as the number of samples increased, which meant adequate sequencing in this experiment (Supplementary Figure 1(b)). Interestingly, four *α*-diversity indices among the three groups were comparable, revealing that ZYD did not significantly affect intrasample species richness and diversity (Supplementary Figure 1(c)).

**Figure 2:**
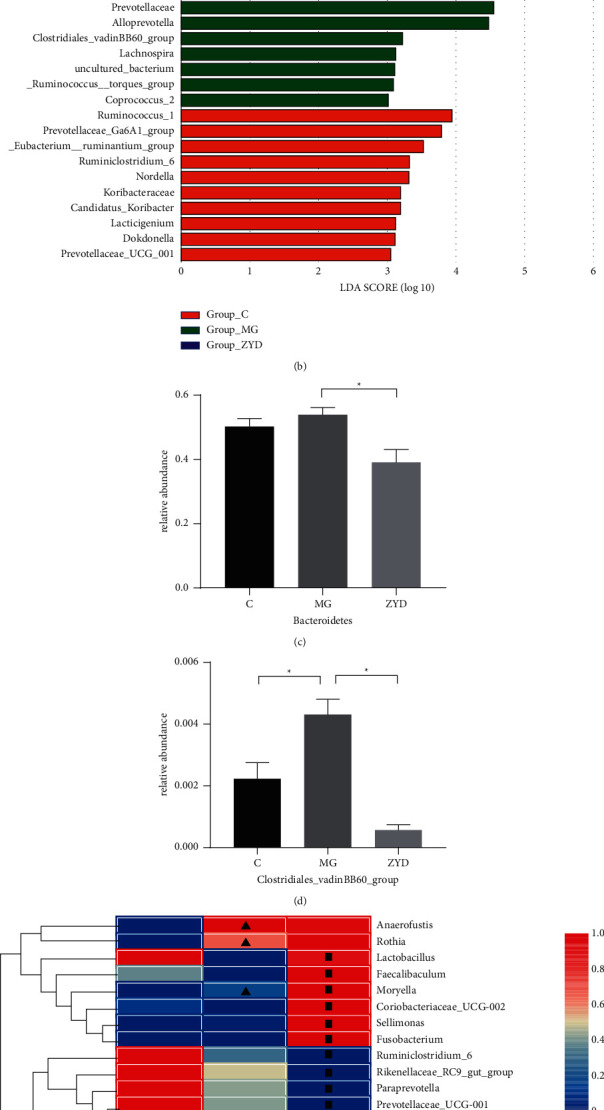
ZYD modulated the gut microbiome in SAP-AKI. (a) Nonmetric multidimensional scaling (NMDS) based on Bray-Curtis distance compared gut microbial structure among the three groups. (b) Linear discriminant analysis (LDA) analysis identified the predominant bacterial taxa among the three groups. (c) ZYD decreased Bacteroidetes (^*∗*^: *P* < 0.05, Mann-Whitney *U* test). *n* = 7 (per group). Data are presented as the mean ± SEM. (d) The variation trend of Clostridiales vadin BB60 group (^*∗*^: *P* < 0.05, Mann-Whitney *U* test). *n* = 7 (per group). Data are presented as the mean ± SEM. (e) Heatmap showed the abundance change in 30 genera across the three groups, ▲: C vs. MG, *P* < 0.05, Mann-Whitney test; ■: MG vs. ZYD, *P* < 0.05, Mann-Whitney *U* test. *n* = 7 (per group). Data are presented as the mean ± SEM. (f) KEGG pathway analysis of the gut microbiota functional gene. C: Healthy control group with the sham operation, MG: severe acute pancreatitis model group, and ZYD: Zengye decoction treatment group.

The *β*-diversity analysis (NMDS based on Bray-Curtis distance) displays the similarity of the overall bacterial structure [34]. Although the cluster of samples in MG could not be separated from the C, ZYD dramatically shifted microbial structure from SAP-AKI status (ANOSIM *R* = 0.14, *P*=0.025) (Figure 2(a)). The LDA of effect size (LEfSe) identified 10, 10, and 14 predominant bacterial taxa in the C, MG, and ZYD, respectively (from phyla to genera, LDA > 3, and *P* < 0.05) (Figure 2(b), Supplementary Figure 1(d)), which suggests a different microbial composition among the three groups. In addition, potentially pathogenic bacteria [40], such as *Bacteroidetes*, were over-represented in the MG. At the same time, short-chain fatty acid (SCFA) producers—*Erysipelotrichaceae*, *Bifidobacterium*, and *Lactobacillus*—were predominant in the ZYD [41, 42].

Next, the alterations of microbial compositions were assessed by the Mann-Whitney *U* test. At the phylum level, SAP-AKI increased the abundance of *Bacteroidetes*, but ZYD decreased it (*P* < 0.05) (Figure 2(c)). At the family level, the Clostridiales vadin BB60 group was increased by SAP-AKI (*P* < 0.05) but was decreased by ZYD (*P* < 0.05) (Figure 2(d)). At the genus level, 12 genera were shifted by SAP-AKI status (*P* < 0.05), and 20 genera were regulated by ZYD (*P* < 0.05) (Figure 2(e)). Notably, ZYD increased *Lactobacillus* (*P* < 0.05) and *Moryella* (*P* < 0.05) while decreasing uncultured_Clostridiales_bacterium (*P* < 0.05). Collectively, these results pointed to a significant modulation effect by ZYD treatment on the gut microbial profile.

Furthermore, KEGG pathway analysis showed that the gut microflora functional gene of amino acid metabolism was more abundant in the ZYD group, implying that the amino acid metabolome may be regulated by ZYD (Figure 2(f)).

### 3.3. ZYD Regulated the Serum Amino Acid Metabolome in SAP-AKI

**Figure 3:**
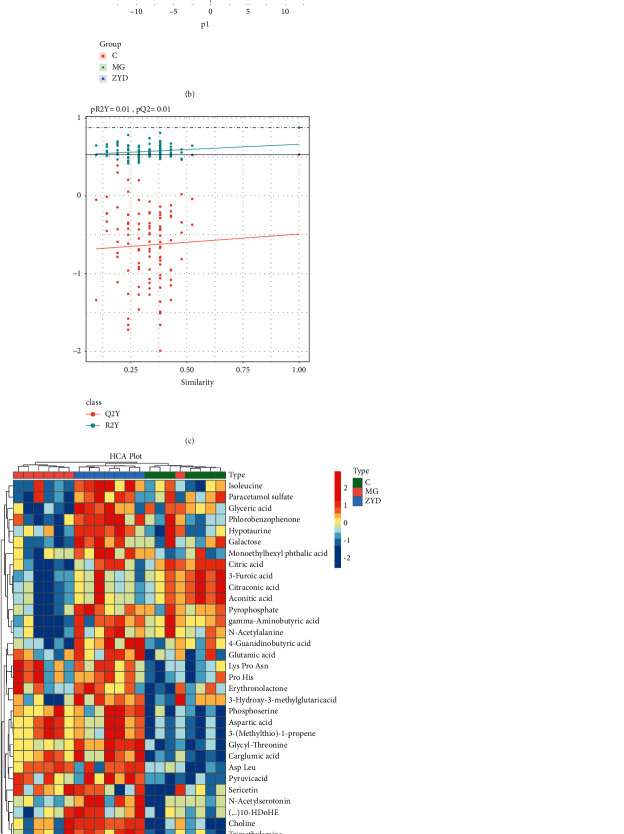
ZYD regulated the serum amino acid metabolome. (a) Principal component analysis (PCA) of the amino acid metabolites among the three groups. (b) The partial least squares-discriminant analysis (PLS-DA) compared the metabolic profile across groups. (c) Permutation test of this PLS-DA model. (d) The heatmap and hierarchical clustering analysis (HCA) detailed the variation of the three groups' 53 differential metabolites (Kruskal-Wallis test, *P* < 0.05). The correlation of samples was decided by Euclidean distance. (e) KEGG pathway enrichment analysis of the 53 differential metabolites. C: Healthy control group with the sham operation, MG: severe acute pancreatitis model group, and ZYD: Zengye decoction treatment group. *n* = 7 (per group).

To explore the effect of ZYD on the serum amino acid metabolome, we performed the metabolomic analysis by LC-MS/MS. Totally, 814 metabolites were identified from the 21 serum samples. Unsupervised method PCA presented the variation trend in the data and detected the potential outlier of serum samples [43]. The PCA scatter plot (Figure 3(a)) showed that the first principal component (PC1) covered 14.59% of the variation and separated the SAP-AKI status and healthy control group (except one outlier in the MG). The second principal component (PC2) covered 12.68% of the variation and distinctly divided the MG and ZYD group. Furthermore, the supervised method PLS-DA was applied to characterize the global metabolic difference across groups [43]. According to the group, distinguished clusters were shaped in the PLS-DA plot, indicating that the metabolic phenotype was dramatically changed by SAP-AKI and ZYD (Figure 3(b)). Correspondingly, permutation test showed a good interpretability and predictability of this PLS-DA model (R2Y 0.878, *P* = 0.01; Q2 0.531, *P*=0.01) (Figure 3(c)) [43]. Thus, the subsequent analysis could be implemented.

The 53 metabolites with statistical significance across groups were selected by Kruskal-Wallis test (*P* < 0.05) and variable importance for the projection score (VIP >1). HCA is an effective algorithm to sort similar samples based on the relative areas of characteristic peaks detected by LC-MS/MS [44]. Combining the HCA and heatmap visualization helps discover the variation trend of these differential metabolites [45].  Figure 3(d) presented a remarkable abundance change in these metabolites after SAP-AKI modeling and ZYD treatment. Plus, twenty-one serum samples were clustered into three categories (except one outlier), and the cluster of ZYD was closer to the control group than MGs. It can be inferred that ZYD can regulate the disturbed metabolic profile of amino acids in SAP-AKI.

To explore the underlying protective mechanism of ZYD, we next performed the KEGG pathway enrichment analysis of these metabolites. As the bubble chart showed, multiple pathways were enriched by these metabolites (Figure 3(e)). Among them, the KEGG pathway of alanine, aspartate, and glutamate metabolism (*P* < 0.01, Impact = 0.5) may play a critical role during the ZYD therapy for SAP-AKI. These results collectively presented a remarkable change in amino acid metabolome after SAP-AKI induction and an apparent regulation by ZYD treatment.

### 3.4. Correlation between the Differential Genera and Metabolites

**Figure 4:**
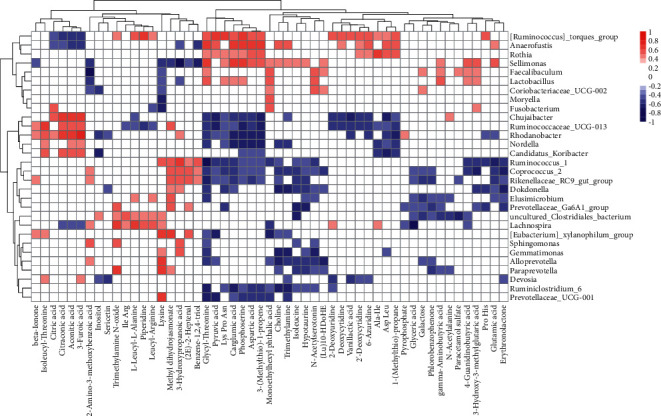
Correlation between the gut microbiota and serum metabolites. The heatmap detailed the positive (red) and negative (blue) correlation between the differential genera (row) and serum amino acid metabolites (column) across groups. This significant correlation was decided by Spearman's correlation analysis (absolute *r* value > 0.4 and FDR *P* < 0.2).

Spearman's correlation analysis was performed on the 30 genera and 53 metabolites with a statistical difference (Kruskal-Wallis test, *P* < 0.05) across the three groups. Interestingly, significant interactions were identified (absolute *r* > 0.4 and FDR *P* < 0.2) among these genera and metabolites, indicating that gut bacteria likely induced the change in serum metabolite level (Figure 4).

## 4. Discussion

SAP-AKI raises the risk of developing chronic kidney disease and the mortality of patients, but the exact pathological mechanism remains unclear and unique treatments are urgently needed [46]. This study successfully established the SAP-AKI rat model and observed a disturbance in the gut microbiome and serum metabolome. Our results presented that ZYD reshaped the landscape of the gut microflora, conferred resistance to amino acid metabolic imbalance, and showed a protective effect against SAP-AKI.

Secretion of amylase and lipase is an important function of pancreatic acinar cells. When pancreatic acinar cells are damaged, a mass of amylase and lipase will directly enter the circulatory system rather than the digestive duct [47, 48]. Thus, amylase and lipase are the globally recommended biomarkers for assessing acute pancreatitis severity [49]. In this study, the significant increase in both indices and histopathological scores suggested a successful SAP model establishment. As expected, ZYD decreased serum amylase and lipase to a large extent, though not reached statistical significance, which also implied the protective effect of ZYD to pancreatic acinar cells during SAP. This speculation was also supported by our histopathological results, in which the pathological injury of acinar cells was obviously reduced by ZYD treatment. At present, the diagnosis of AKI complicating from SAP depends on the dynamic increase in serum Cr [49]. BUN is usually used to evaluate kidney function in clinical practice [50]. However, both do not accurately reflect kidney injury severity, especially in early-stage SAP [51]. Therefore, the changes in Cr and BUN in this study were slight and insignificant. One preliminary study proved the diagnostic value of urinary KIM-1 concentration for SAP-AKI patients, which showed the shortcomings like lasting a short time and the need for frequent monitoring [52]. A rare study explored the serum KIM-1 value for SAP-AKI severity assessment [51]. But Chang et al. discovered a significant increase in serum KIM-1 in the hemorrhagic shock rat model with AKI [53]. Consistent with the literature, we found that serum KIM-1 was significantly lifted in the 36 h SAP-AKI rat model but approached normal after ZYD treatment. So, this result suggests the diagnostic value of serum KIM-1 for SAP-AKI in the early stage. More research studies are needed to elucidate this point.

Emerging research indicated that gut microbiota plays an essential role in the progression of acute pancreatitis [45, 46], implying that modulating gut microbial structure could be an efficient therapy for SAP. An earlier study reported that chitosan oligosaccharides attenuated SAP by modulating the *β*-diversity and predominant composition of gut bacteria [54]. Partly in line with previous research [9], ZYD also showed the capability to shift the gut bacterial structure from SAP-AKI, as evidenced by NMDS and LDA-LEfSe. Thus, we speculate that ZYD improves SAP-AKI via the modulation of gut microflora. The *Bacteroidetes* (phylum), potentially pathogenic bacteria [55], were a dominant gut bacterial member in the SAP-AKI. Similarly, the Clostridiales vadin BB60 group (family) and uncultured_Clostridiales_bacterium (genus), both belonging to the opportunistically pathogenic Clostridiales (order) [56], significantly increased in the SAP-AKI status. However, our results demonstrated that these pathogenic bacteria were effectively decreased by ZYD treatment. So, it can be inferred that reducing pathogenic microbiota may be closely related to the protective mechanism of ZYD. Alteration of gut microbial composition contributes to the variation of bacterial metabolites, which may influence the progression of SAP [57]. SCFAs, a widely studied metabolite of bacteria, showed good performance in maintaining intestinal homeostasis [58]. Research studies also indicated that supplements of SCFAs helped ameliorate organ injuries in SAP [59, 60]. In this study, SCFA-producing strains—*Erysipelotrichaceae*, *Bifidobacterium*, *Lactobacillus*, and *Moryella*, were more abundant in ZYD than MG [42, 61]. It suggests that ZYD protects SAP-AKI by increasing these bacteria to indirectly supplement SCFAs. In a word, our results imply that ZYD ameliorates SAP-AKI by modulating the gut microbiome, including reducing pathogenic bacteria and improving SCFA-producing strains.

Amino acids are a substantial energy source to fuel the body [62]. In acute pancreatitis, systemic inflammation causes a hypercatabolic state, contributing to increased energy requirements and disrupting the metabolism of amino acids [13, 63]. For critical patients with AKI, the hypercatabolic condition also negatively affects protein degradation and amino acid conversions [64]. So, regulating the metabolism of amino acids may serve as a potential therapy for SAP-AKI. The previous study has elucidated the capability of Chinese medicine to treat acute pancreatitis by altering the metabolic profile [16]. As expected, the unique metabolic phenotypes of amino acids in SAP-AKI and after ZYD treatment are presented in the scatter plot of PCA and PLS-DA. Moreover, the variation of the 53 differential metabolites across groups revealed that the ZYD could alter the metabolic profile of amino acids toward healthy status. In short, these results imply that ZYD attenuates SAP-AKI by regulating the amino acid metabolome. One unexpected finding was an outlier from the MG group, but we considered it due to a lesser degree of illness after SAP-AKI modeling. In addition, KEGG analysis demonstrated that ZYD might affect multiple pathways to therapy for SAP-AKI, but the way of alanine, aspartate, and glutamate metabolism significantly enriched by multiple metabolites is worth great attention. Alanine, aspartate, and glutamate play an essential role in protein structure and energy supplement through the tricarboxylic cycle [62]. Our result showed that SAP bothered the metabolism of these metabolites in this pathway, and ZYD significantly regulated most of them and notably increased glutamic acid (glutamate). The supply of glutamine, which can be hydrolyzed to glutamate, could improve gut permeability, oxidative stress, and reduce the complication rate in SAP patients [65]. Thus, it can be inferred that ZYD could regulate energy supplements by influencing the pathway of alanine, aspartate, and glutamate metabolism to protect SAP-AKI, but more research studies are needed to elucidate it.

The metabolome is responsive to the physiological condition and gut microflora variation [66, 67]. Amino acids have emerged as critical signaling metabolites to regulate metabolism and inflammation through the relationship with microbiota and host receptors [68]. Correspondingly, in this study, significant correlations were identified between gut bacteria and amino acids, which were also modulated by SAP-AKI and ZYD. Therefore, we speculate that the interaction of gut microbiota and serum metabolome was related to the underlying mechanism of ZYD protection.

There are several limitations to this study. First, these experimental data come from a small number of SAP-AKI animal models. Second, the current developing level of the methodology may limit the detection of the gut microbiome and metabolome in serum. However, this study provided a new therapy to SAP-AKI and preliminary elucidated the underlying mechanism. Our findings may be helpful for further comprehension of the SAP-AKI pathology and appropriate clinical application for ZYD.

## 5. Conclusions

ZYD attenuated SAP-AKI by modulating the gut microbiome and serum amino acid metabolome, which may be a promising adjuvant treatment.

## Data Availability

The datasets used and/or analyzed during the current study are available from the corresponding author upon reasonable request.

## Supplementary Materials

Supplementary Materials The supplementary figures were uploaded with the original manuscript.
