# Supplementary material for: Zengye Decoction Attenuated Severe Acute Pancreatitis Complicated with Acute Kidney Injury by Modulating the Gut Microbiome and Serum Amino Acid Metabolome
Source: Evid Based Complement Alternat Med. 2022 May 9;2022:1588786. doi: 10.1155/2022/1588786 (PMC9110161; doi:10.1155/2022/1588786)
Supplement: Supplementary Materials — The supplementary figures were uploaded with the original manuscript. [file 1588786.f1.zip › 1588786.f1/ARRIVE guidelines 2.0 - English.pdf]

National Centre
for the Replacement
Refinement & Reduction
of Animals in Research

The ARRIVE guidelines 2.0

Animal Research: Reporting of *In Vivo* Experiments

Nathalie Percie du Sert[1], Viki Hurst[1], Amrita Ahluwalia[2], Sabina Alam[3], Marc T Avey[4], Monya Baker[5], William J Browne[6], Alejandra Clark[7], Innes C Cuthill[6], Ulrich Dirnagl[8], Michael Emerson[9], Paul Garner[10], Stephen T Holgate[11], David W Howells[12], Natasha A Karp[13], Stanley E Lazic[14], Katie Lidster[1], Catriona J MacCallum[15], Malcolm Macleod[16], Esther J Pearl[1], Ole H Petersen[17], Frances Rawle[18], Penny Reynolds[19], Kieron Rooney[20], Emily S Sena[16], Shai D Silberberg[21], Thomas Steckler[22], Hanno Würbel[23]

[1]NC3Rs, UK. [2]Queen Mary University of London, UK. [3]Taylor & Francis Group, UK. [4]ICF, USA. [5]Nature, USA. [6]University of Bristol, UK. [7]PLOS ONE, UK. [8]Charite Universitätsmedizin Berlin, Germany. [9]Imperial College London, UK. [10]Liverpool School of Tropical Medicine, UK. [11]University of Southampton, UK. [12]University of Tasmania, Australia. [13]AstraZeneca, UK. [14]Prioris.ai Inc, Canada [15]Hindawi Ltd, UK. [16]University of Edinburgh, UK. [17]Cardiff University, UK. [18]Medical Research Council, UK. [19]University of Florida, USA. [20]University of Sydney, Australia. [21]National Institute of Neurological Disorders and Stroke, USA. [22]Janssen Pharmaceutica NV, Belgium. [23] Universität Bern, Switzerland

The ARRIVE guidelines 2.0 are a checklist of information to include in publications describing animal research, published in *PLOS Biology* in July 2020. They ensure that studies are reported in enough detail to add to the knowledge base. This transparency enables readers and reviewers to scrutinise the research adequately, evaluate its methodological rigour, and reproduce the methods or findings.

Improving transparency in animal research – why ARRIVE?

Issues around the reproducibility of research involving animals cause significant concern among scientists, funders, and policy makers.

Transparent and accurate reporting is a cornerstone of reproducibility. It allows the research to be assessed effectively so it can inform future research, policy, and clinical practice.

However, animal research publications often lack important information, which prevents adequate evaluation of the methods and findings. To address this, the ARRIVE guidelines were first published in 2010. The updated guidelines – ARRIVE 2.0 – were released in 2020, along with a separate Explanation and Elaboration document providing further context.

Introducing ARRIVE 2.0

ARRIVE 2.0 is the result of an extensive, international collaboration, with input from the scientific community carefully built into the process. The authors of the guidelines include funders, journal editors, methodologists, statisticians, and researchers from academia and industry. Additional input from external stakeholders was gathered via a Delphi exercise. The guidelines were also road-tested by researchers preparing manuscripts, to ensure that they are well-understood and useful in practice.

The guidelines are relevant to any study involving live animals, from mammals to fish, as well as invertebrates, in any area of the biosciences.

To allow for initial focus on the most critical issues, the items which make up the guidelines are classified into two prioritised sets, with no ranking within each set. Both are provided overleaf. Reporting the items in both sets represents best practice.

How to use the guidelines

The guidelines are useful to consult throughout the course of a study:

- **During study planning:** the guidelines and accompanying Explanation and Elaboration document provide recommendations on experimental design, minimisation of bias, sample size and statistical analyses, helping researchers design rigorous and reliable *in vivo* experiments.
- **During the conduct of a study:** this allows researchers to record important information about study methods, which will be needed later for manuscript preparation.
- **When writing a manuscript:** used as an *aide memoire* to ensure the manuscript contains all relevant information.
- **When reviewing a manuscript:** to ensure all relevant information is available to evaluate the research.

Resources to support the use of ARRIVE 2.0

A wide range of resources are available at www.ARRIVEguidelines.org. These include:

- **Explanation and Elaboration for each of the items in the guidelines.** This includes extensive advice on the design of animal experiments, provides the rationale and evidence behind each item in the guidelines, and gives clear examples of good reporting from the published literature.
- **Fillable ARRIVE 2.0 checklists.** This allows researchers to indicate the specific sections of a manuscript that contain information relating to each item. Checklists are available for the ARRIVE Essential 10 and for the full ARRIVE 2.0 so that journals can tailor their requirements.
- **ARRIVE supporters.** This includes information on how journals, funders, institutions and other organisations can use and promote the guidelines.
- **The guidelines are available in multiple languages.** This helps international uptake.

Where to find the guidelines

Percie du Sert N, Hurst V, Ahluwalia A *et al.* (2020). The ARRIVE guidelines 2.0: updated guidelines for reporting animal research. *PLOS Biology*. doi: 10.1371/journal.pbio.3000410

Percie du Sert N, Ahluwalia A, Alam S *et al.* (2020). Reporting animal research: Explanation and Elaboration for the ARRIVE guidelines 2.0. *PLOS Biology*. doi: 10.1371/journal.pbio.3000411

Acknowledgements

We are grateful to the members of the expert panel who took part in the Delphi exercise during the development of these guidelines, and the participants in the road testing for their time and feedback.

Further information

www.ARRIVEguidelines.org
arrive@nc3rs.org.uk
@NC3Rs

The ARRIVE Essential 10

These items are the basic minimum to include in a manuscript. Without this information, readers and reviewers cannot assess the reliability of the findings.

**Study design**	1	For each experiment, provide brief details of study design including:<br>a. The groups being compared, including control groups. If no control group has been used, the rationale should be stated.<br>b. The experimental unit (e.g. a single animal, litter, or cage of animals).	No.4
**Sample size**	2	a. Specify the exact number of experimental units allocated to each group, and the total number in each experiment. Also indicate the total number of animals used.<br>b. Explain how the sample size was decided. Provide details of any *a priori* sample size calculation, if done.	No.4
**Inclusion and exclusion criteria**	3	a. Describe any criteria used for including and excluding animals (or experimental units) during the experiment, and data points during the analysis. Specify if these criteria were established *a priori*. If no criteria were set, state this explicitly.<br>b. For each experimental group, report any animals, experimental units or data points not included in the analysis and explain why. If there were no exclusions, state so.<br>c. For each analysis, report the exact value of *n* in each experimental group.	Not Applicable
**Randomisation**	4	a. State whether randomisation was used to allocate experimental units to control and treatment groups. If done, provide the method used to generate the randomisation sequence.<br>b. Describe the strategy used to minimise potential confounders such as the order of treatments and measurements, or animal/cage location. If confounders were not controlled, state this explicitly.	No.4
**Blinding**	5	Describe who was aware of the group allocation at the different stages of the experiment (during the allocation, the conduct of the experiment, the outcome assessment, and the data analysis).	No.4
**Outcome measures**	6	a. Clearly define all outcome measures assessed (e.g. cell death, molecular markers, or behavioural changes).<br>b. For hypothesis-testing studies, specify the primary outcome measure, i.e. the outcome measure that was used to determine the sample size.	No.4-6
**Statistical methods**	7	a. Provide details of the statistical methods used for each analysis, including software used.<br>b. Describe any methods used to assess whether the data met the assumptions of the statistical approach, and what was done if the assumptions were not met.	No.6
**Experimental animals**	8	a. Provide species-appropriate details of the animals used, including species, strain and substrain, sex, age or developmental stage, and, if relevant, weight.<br>b. Provide further relevant information on the provenance of animals, health/immune status, genetic modification status, genotype, and any previous procedures.	No.3
**Experimental procedures**	9	For each experimental group, including controls, describe the procedures in enough detail to allow others to replicate them, including:<br>a. What was done, how it was done and what was used.<br>b. When and how often.<br>c. Where (including detail of any acclimatisation periods).<br>d. Why (provide rationale for procedures).	No.4
**Results**	10	For each experiment conducted, including independent replications, report:<br>a. Summary/descriptive statistics for each experimental group, with a measure of variability where applicable (e.g. mean and SD, or median and range).<br>b. If applicable, the effect size with a confidence interval.	No.4

The Recommended Set

These items complement the Essential 10 and add important context to the study. Reporting the items in both sets represents best practice.

**Abstract**	11	Provide an accurate summary of the research objectives, animal species, strain and sex, key methods, principal findings, and study conclusions.	No.2
**Background**	12	a. Include sufficient scientific background to understand the rationale and context for the study, and explain the experimental approach.<br>b. Explain how the animal species and model used address the scientific objectives and, where appropriate, the relevance to human biology.	No.3
**Objectives**	13	Clearly describe the research question, research objectives and, where appropriate, specific hypotheses being tested.	No.3
**Ethical statement**	14	Provide the name of the ethical review committee or equivalent that has approved the use of animals in this study, and any relevant licence or protocol numbers (if applicable). If ethical approval was not sought or granted, provide a justification.	No.3
**Housing and husbandry**	15	Provide details of housing and husbandry conditions, including any environmental enrichment.	No.3
**Animal care and monitoring**	16	a. Describe any interventions or steps taken in the experimental protocols to reduce pain, suffering and distress.<br>b. Report any expected or unexpected adverse events.<br>c. Describe the humane endpoints established for the study, the signs that were monitored and the frequency of monitoring. If the study did not have humane endpoints, state this.	No.3
**Interpretation/ scientific implications**	17	a. Interpret the results, taking into account the study objectives and hypotheses, current theory and other relevant studies in the literature.<br>b. Comment on the study limitations including potential sources of bias, limitations of the animal model, and imprecision associated with the results.	No.10
**Generalisability/ translation**	18	Comment on whether, and how, the findings of this study are likely to generalise to other species or experimental conditions, including any relevance to human biology (where appropriate).	No.10
**Protocol registration**	19	Provide a statement indicating whether a protocol (including the research question, key design features, and analysis plan) was prepared before the study, and if and where this protocol was registered.	No.3
**Data access**	20	Provide a statement describing if and where study data are available.	No.11
**Declaration of interests**	21	a. Declare any potential conflicts of interest, including financial and non-financial. If none exist, this should be stated.<br>b. List all funding sources (including grant identifier) and the role of the funder(s) in the design, analysis and reporting of the study.	No.11

The ARRIVE guidelines 2.0: updated guidelines for reporting animal research. Originally published in *PLOS Biology*, July 2020.
