# Supplementary material for: Zengye Decoction Attenuated Severe Acute Pancreatitis Complicated with Acute Kidney Injury by Modulating the Gut Microbiome and Serum Amino Acid Metabolome
Source: Evid Based Complement Alternat Med. 2022 May 9;2022:1588786. doi: 10.1155/2022/1588786 (PMC9110161; doi:10.1155/2022/1588786)
Supplement: Supplementary Materials — The supplementary figures were uploaded with the original manuscript. [file 1588786.f1.zip › 1588786.f1/Legend for Supplementary Figure 1.docx]

**Supplementary** **Figure 1**
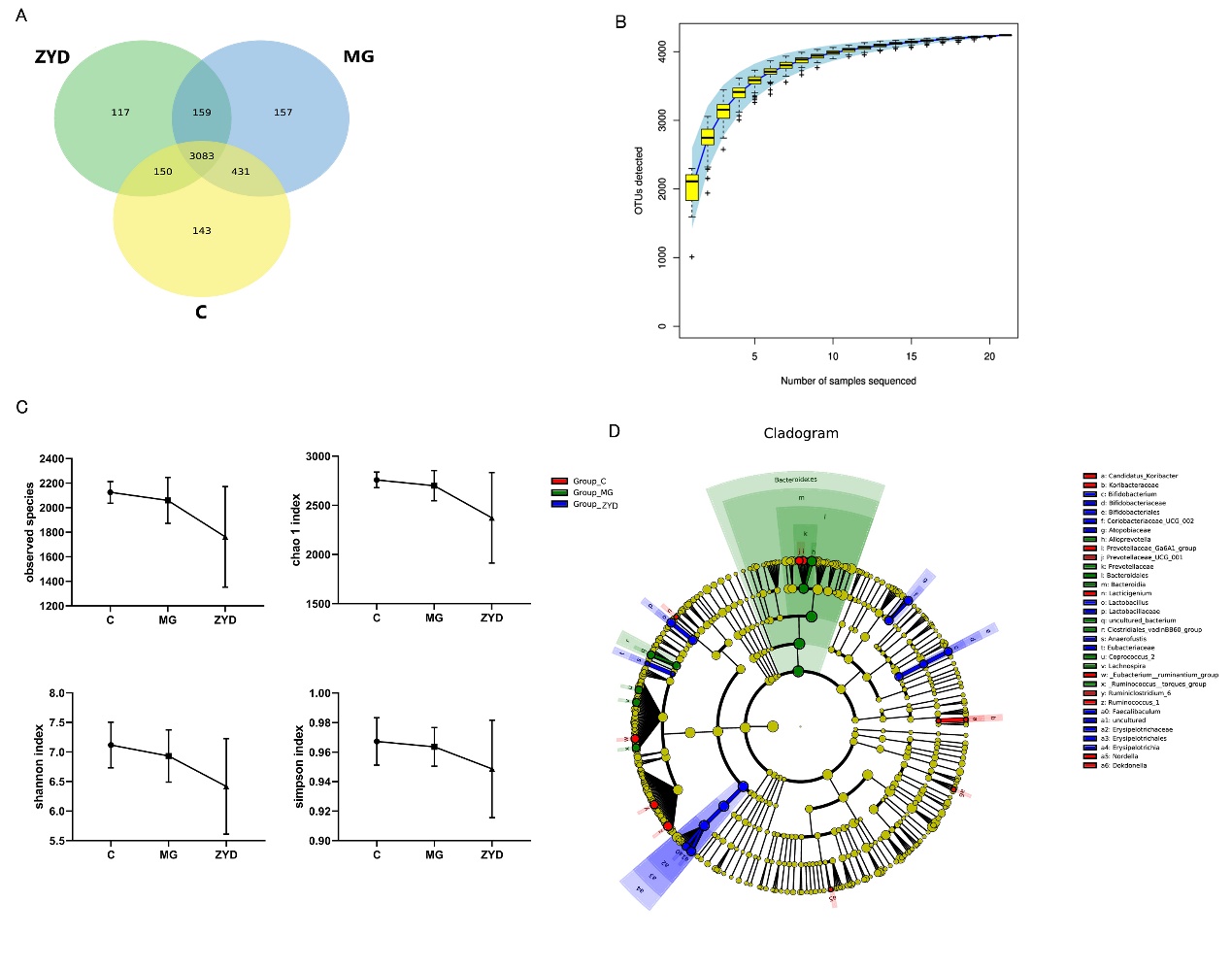
**: A:** The number of operational taxonomic units (OTUs) detected among the three groups. **B:** The species accumulation curves showed adequate sequencing in this experiment. **C:** Zengye decoction did not significantly affect the α diversity indices. n=7 (per group). Data are presented as the mean ± SEM. **D:** The Linear discriminant analysis of effect size (LEfSe) identified 10, 10, 14 predominant bacterial taxa (from the phylum level to the genus level) in the C, MG, and ZYD, respectively. C: healthy control group with sham-operation, MG: severe acute pancreatitis model group, and ZYD: Zengye decoction treatment group.
