# Supplementary material for: Zengye Decoction Attenuated Severe Acute Pancreatitis Complicated with Acute Kidney Injury by Modulating the Gut Microbiome and Serum Amino Acid Metabolome
Source: Evid Based Complement Alternat Med. 2022 May 9;2022:1588786. doi: 10.1155/2022/1588786 (PMC9110161; doi:10.1155/2022/1588786)
Supplement: Supplementary Materials — The supplementary figures were uploaded with the original manuscript. [file 1588786.f1.zip › 1588786.f1/supplementary Figure 1D.pdf]

Cladogram

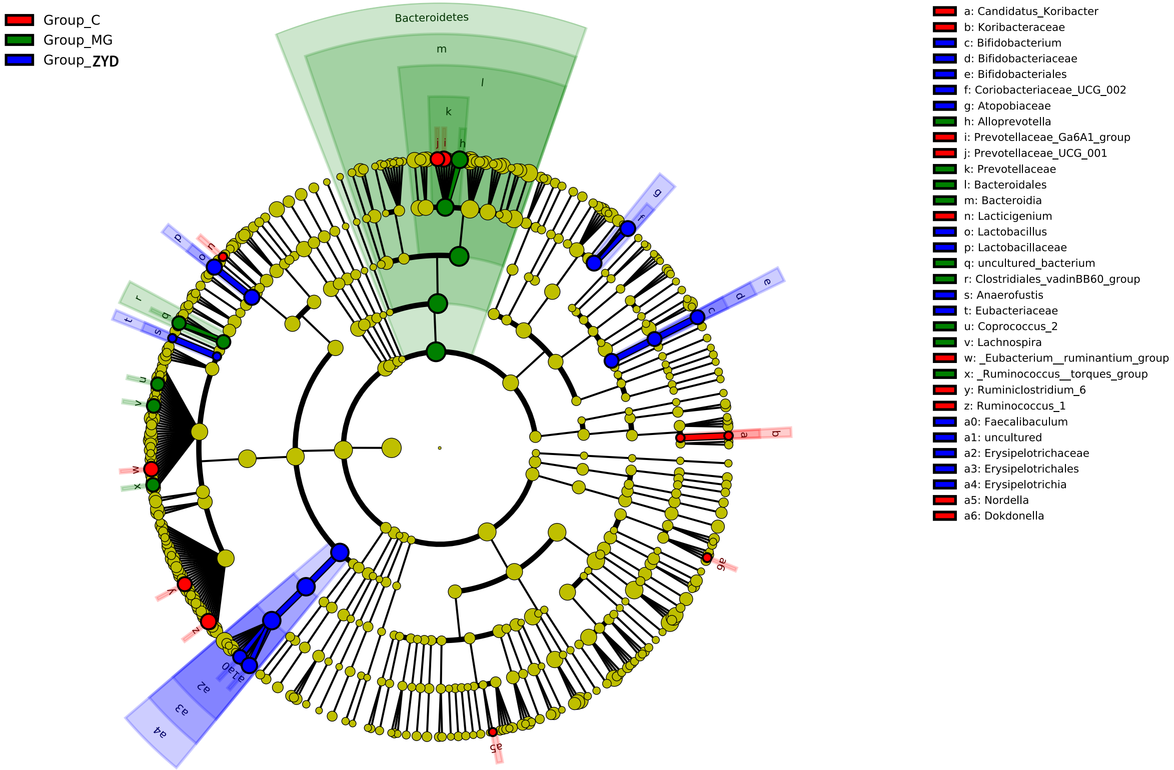
Group_C
Group_MG
Group_ZYD
Bacteroidetes
a: Candidatus_Koribacter
b: Koribacteraceae
c: Bifidobacterium
d: Bifidobacteriaceae
e: Bifidobacteriales
f: Coriobacteriaceae_UCG_002
g: Atopobiaceae
h: Alloprevotella
i: Prevotellaceae_Ga6A1_group
j: Prevotellaceae_UCG_001
k: Prevotellaceae
l: Bacteroidales
m: Bacteroidia
n: Lacticigenium
o: Lactobacillus
p: Lactobacillaceae
q: uncultured_bacterium
r: Clostridiales_vadinBB60_group
s: Anaerofustis
t: Eubacteriaceae
u: Coprococcus_2
v: Lachnospira
w: _Eubacterium__ruminantium_group
x: _Ruminococcus__torques_group
y: Ruminiclostridium_6
z: Ruminococcus_1
a0: Faecalibaculum
a1: uncultured
a2: Erysipelotrichaceae
a3: Erysipelotrichales
a4: Erysipelotrichia
a5: Nordella
a6: Dokdonella
