# Supplementary figures and images for: Zengye Decoction Attenuated Severe Acute Pancreatitis Complicated with Acute Kidney Injury by Modulating the Gut Microbiome and Serum Amino Acid Metabolome
Source: Evid Based Complement Alternat Med. 2022 May 9;2022:1588786. doi: 10.1155/2022/1588786 (PMC9110161; doi:10.1155/2022/1588786)

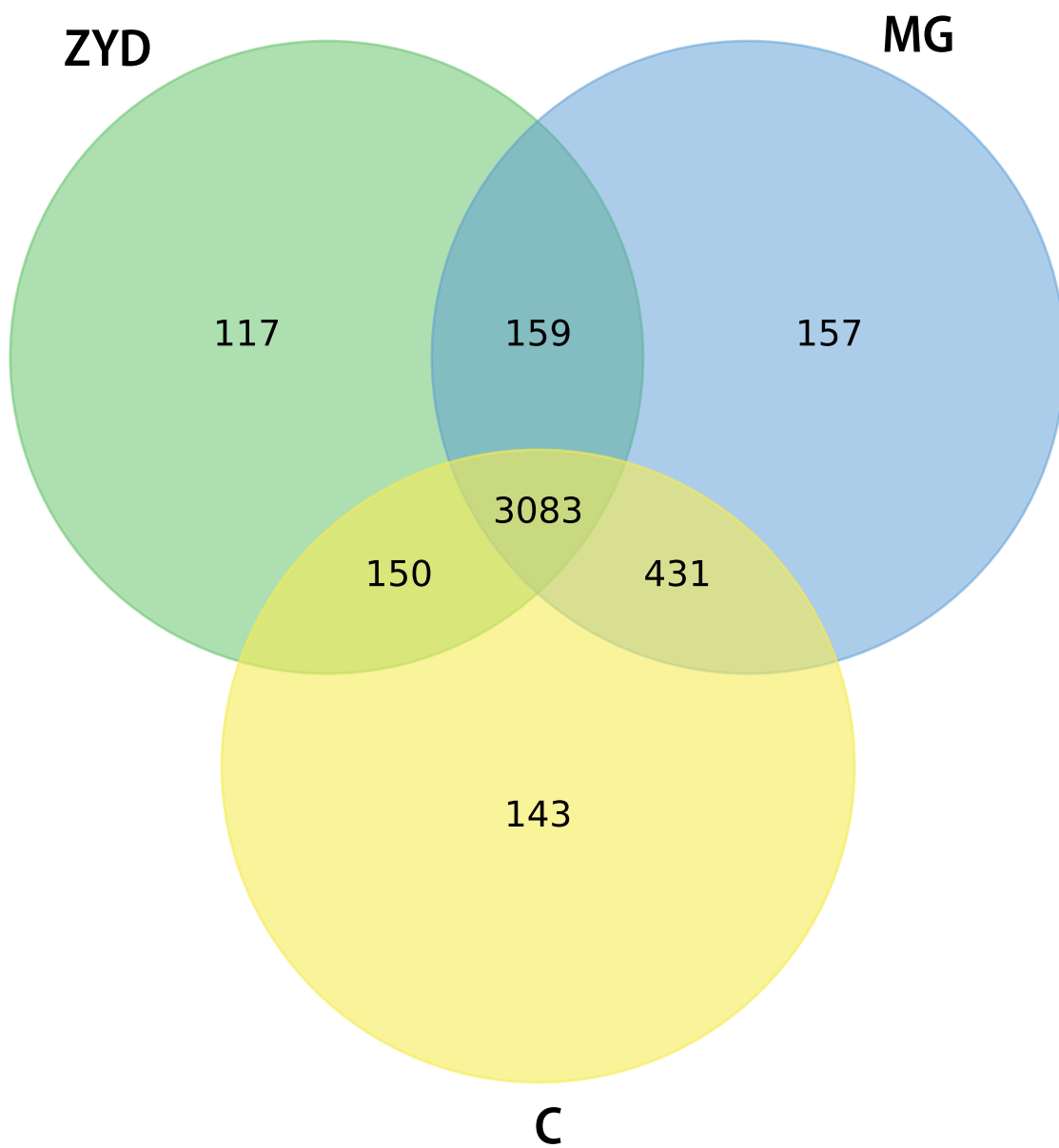

Supplement: Supplementary Materials — The supplementary figures were uploaded with the original manuscript. [file 1588786.f1.zip › 1588786.f1/supplementary Figure 1A.pdf]

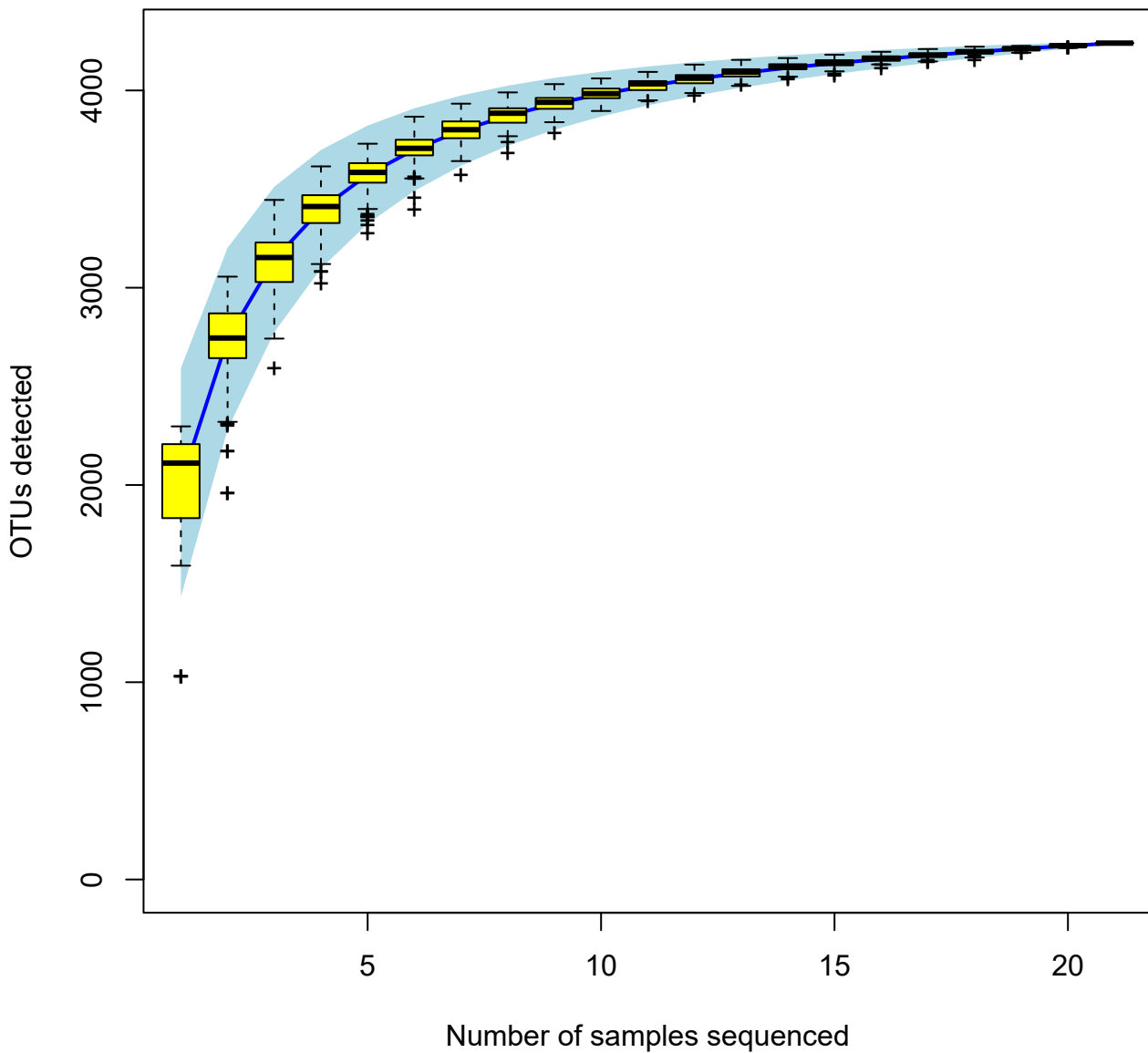

Supplement: Supplementary Materials — The supplementary figures were uploaded with the original manuscript. [file 1588786.f1.zip › 1588786.f1/supplementary Figure 1B.pdf]

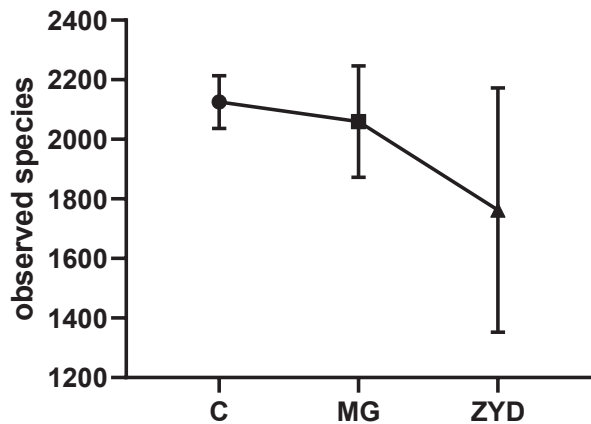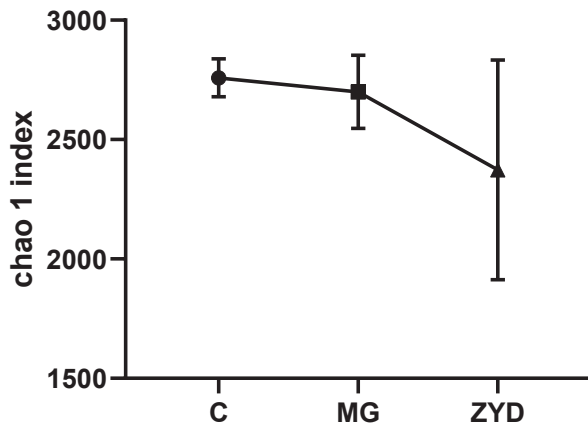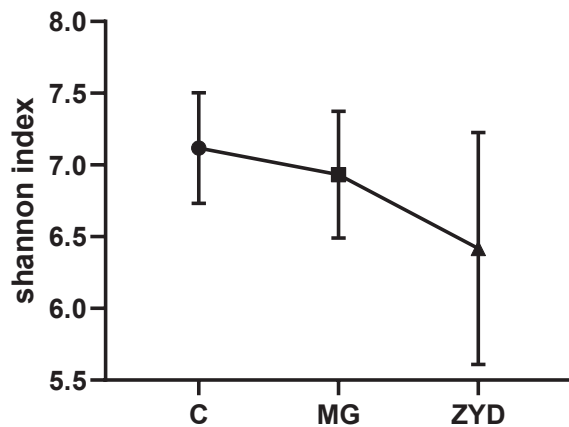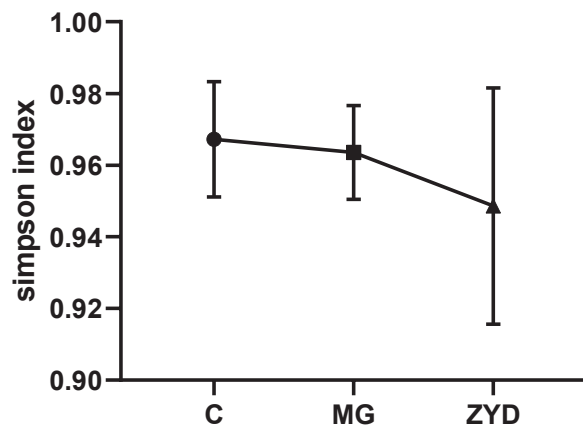

Supplement: Supplementary Materials — The supplementary figures were uploaded with the original manuscript. [file 1588786.f1.zip › 1588786.f1/supplementary Figure 1C.pdf]
